# Vulvar Leiomyosarcoma Masquerading as a Bartholin’s Gland Cyst in an Adolescent

**DOI:** 10.7759/cureus.21674

**Published:** 2022-01-27

**Authors:** Trenton Reinicke, Danyon J Anderson, Devesh Kumar, Cornelia Griggs

**Affiliations:** 1 Department of Clinical and Translational Epidemiology, Massachusetts General Hospital, Boston, USA; 2 School of Medicine, Medical College of Wisconsin, Wauwatosa, USA; 3 Department of Ophthalmology, Medical College of Wisconsin, Wauwatosa, USA; 4 Department of Surgery, Massachusetts General Hospital, Harvard Medical School, Boston, USA

**Keywords:** bartholin's glands, adolescent medicine, mesenchymal sarcoma, vulva cancer, primary leiomyosarcoma

## Abstract

Sarcomas, tumors of mesenchymal origin, comprise a small percentage of all malignant tumors and are often challenging to diagnose. Leiomyosarcoma (LMS) is a rare form of cancer arising from smooth muscle cells. While a soft tissue sarcoma diagnosis is rare in and of itself, LMS diagnosis at an adolescent age is even more unique. Vulvar LMS can easily be misdiagnosed as a benign vaginal lesion, leading to a delay in proper treatment and poorer outcomes. In this case, we present a 14-year-old female who was diagnosed with a grade 2 vulvar LMS that clinically mimicked a Bartholin’s gland cyst.

## Introduction

Sarcoma refers to a malignant neoplasm formed among mesenchymal tissue. Accounting for 1% of all malignancies, sarcomas are composed of neoplasms arising from bone, cartilage, fat, muscle, blood vessels, and fibrous and connective tissue [1,2]. Leiomyosarcoma (LMS) represents a rare form of cancer that grows within the smooth muscles and is commonly found in the abdomen, retroperitoneum, larger blood vessels, and uterus [3]. While the incidence of soft tissue sarcomas ranges from 20 to 30 per 1,000,000 persons in the United States, the diagnosis of LMS among children is exceedingly rare, accounting for only 2% of all adolescent soft tissue sarcomas [4,5]. LMS represents the most common type of vaginal sarcoma among the adult population, but much less is known about LMS among children [6,7]. Furthermore, LMS of the vulva is very rare in any age group, and diagnosis can often be delayed as this form of sarcoma can easily be mistaken for a cyst of the Bartholin’s glands [8,9]. Here, we present the case of a 14-year-old female diagnosed with a primary grade 2 leiomyosarcoma of the vulva that clinically mimicked a left Bartholin’s gland cyst, as well as the associated interventions leading to a successful diagnosis and treatment.

## Case presentation

A 14-year-old Hispanic female presented to her pediatrician for a well-child check with concerns for a mass that could be felt in the vaginal area when sitting in certain positions and that has been growing over time. The patient had reports of rare mild pain in the vaginal area for approximately 10 months prior to the initial visit. Nearly four months later, the physical examination identified an approximately 3 × 2 cm palpable mass in the inferior aspect of the left labia and the edge of the introitus. No discomfort was felt with pressure to the mass. The patient does menstruate on a regular cycle and has never noticed or been diagnosed with any lesions or cysts elsewhere on her body. The mass did not have any open spontaneous drainage. The patient was recommended and scheduled to have surgical drainage and partial excision of the mass following this visit.

Significant medical and surgical history includes the patient being a nonsmoker, not sexually active, not on any medications, and a diagnosis of pediatric obesity with a body mass index (BMI) in the 95th-98th percentile for her age at the time. The patient underwent an appendectomy at the age of eight due to perforated appendicitis, which gave rise to an intraabdominal septic infection with the development of abdominal abscesses, which was successfully treated after a prolonged hospitalization. Family medical history includes the patient’s mother being positive for purified protein derivative (PPD), but no other significant family medical history is reported. Upon examination at the well-child check, the patient’s pediatrician noted a 2 cm non-tender, round, mobile swelling at the left posterior opening of the vagina. This finding was believed to be an uncomplicated Bartholin’s cyst without abscess or infection, and the patient was referred to pediatric surgery for additional evaluation and management.

Approximately one month following the pre-operation appointment, the patient underwent drainage and partial excision of the presumed left Bartholin’s cyst. The patient and her parents have continued to deny any change in medical history in the interim, including no reported fevers, night sweats, chills, cough, chest pain, shortness of breath, abdominal pain, nausea, vomiting, changes in bowel habits, dysuria, hematuria, urinary frequency, and urinary urgency. During the procedure, the mass was noted at 6 × 6 cm and thought to be a Bartholin’s cyst in the left labia, although it had suspicious solid components at the time of resection. As such, instead of marsupialization or drainage, the surgical team proceeded with the excision of the mass. There was minimal drainage from the mass, which appeared to have multiple lobulations. The mass outside the hymenal ring on the vaginal mucosa was excised using sharp and blunt dissection, and a biopsy was sent to both surgical and molecular pathology.

The surgical pathology report of the biopsy taken during surgery confirmed a leiomyosarcoma of the vulva, grade 2 of 3. The tumor was found to be composed of plump spindle cells with abundant eosinophilic cytoplasm arranged in fascicles. The mitotic rate was reported to be variable but reached as high as 11 per 10 high-power fields (HPFs). The tumor cells stained strongly and diffusely positive for caldesmon and SMA with rare focal positivity for desmin and EMA. Negative stains were reported for MNF116, AE1.3, Cam5.2, ALK1, ROS1, S100, HMB45, MyoD1, and myogenin. An additional immunohistochemical stain for pan-TRK was negative in the tumor cells, while the expression of ATRX was retained. The molecular pathology report included a solid fusion assay, which is based on targeted ribonucleic acid (RNA). Next-generation sequencing (NGS) using anchored multiplex PCR (AMP) detected no reportable fusion transcripts from the biopsy taken. The patient was then scheduled for further imaging, including pelvic magnetic resonance imaging (MRI), positron emission tomography (PET), and computed tomography (CT) later in the week.

Nearly two weeks later and following the cancer diagnosis, the case was presented at the pediatric sarcoma tumor board, and the patient was referred to gynecologic oncology, as well as plastic surgery. The patient was recommended for a radical hemivulvectomy with the possible need of a flap to close the defect. The patient and her parents were informed of the rationale behind this procedure, given the extremely rare nature and aggressiveness of this form of tumor in children, and they agreed to proceed with the surgery.

The patient subsequently underwent the radical hemivulvectomy of the left vulvar leiomyosarcoma and reconstruction with an advancement flap. Biopsies were taken during this procedure, and final pathology revealed no evidence of malignancy on the left vulvar deep margin. Furthermore, there was no evidence of malignancy on the left vulva medial margin or the left vulvar inferior margin. Postoperative MRI, PET, and CT imaging revealed no evidence of metastatic disease. Following a successful tumor excision and reconstruction, the patient was again presented at the sarcoma tumor board in pediatrics, and the recommendation was made for close surveillance only. Following these procedures, the patient has been doing well as the pain is well controlled, and she is planning to be followed with future imaging and clinic visits on an as-needed basis.

## Discussion

Sarcomas are rare neoplasms of mesenchymal origin that account for less than 1% of all adult cancers and approximately 20% of all pediatric malignant solid tumors [5]. While uterine sarcomas are among the most diagnosed gynecological sarcomas, sarcomas localized in the vulva make up only 1%-3% of all vulvar cancers [5]. Sarcomas of the vulva are often characterized by a high metastatic potential, fast growth rate, and high mortality rate [10,11]. The most common vulvar sarcoma is LMS [1], which is most often diagnosed in women of middle age [11]. While the associated literature has defined one other case of vulvar LMS in a 14-year-old, this represents the youngest age known to exhibit this type of sarcoma [11]. Regarding LMS treatment, the recommended therapeutic approach is complete surgical resection [11]. Given this neoplasm’s high recurrence rate and often a late-stage diagnosis, adjuvant therapy is commonly needed for LMS, such as chemotherapy or additional surgeries plus radiotherapy, which was the case for the other reported vulvar LMS case in a 14-year-old [12-15]. Vulvar sarcomas often manifest as asymptomatic, especially in younger individuals, or present with nonspecific local irritation [11]. The late symptoms of vulvar sarcomas associated with poor prognosis, as is the case with our patient, include pain, bleeding, or ulceration [11]. Misdiagnosis of vulvar LMS is common given the nonspecific clinical manifestations of this form of cancer. Often, this neoplasm is mistaken for Bartholin’s gland cysts or even genitourinary rhabdomyosarcomas, which are known to be more prevalent among children [10,16]. While vulvar LMS is rare in and of itself, vulvar malignancies in the pediatric population are exceptionally rare, and their clinicopathologic profile is not well understood [12,15]. Thus, we present another rare incidence of vulvar LMS, with nonspecific clinical manifestation, in an adolescent and the associated interventions leading to successful identification and resection of this disguised cancer without the need for more adjuvant therapy to this point.

## Conclusions

Arising from smooth muscle, leiomyosarcomas (LMS) are known to be rare and aggressive malignancies associated with distressing clinical outcomes. While the uterus is the most common location for LMS occurrence, adolescent vulvar LMS are exceedingly rare and poorly understood. In this case, we presented a 14-year-old female who was diagnosed with a grade 2 vulvar LMS that clinically mimicked a Bartholin’s gland cyst. As such, raised suspicion is warranted for vulvar lesions with atypical features, given that benign assumptions of Bartholin’s gland masses are more plausible in this adolescent age group.
